# Insight into the Digital Health System of Ukraine (eHealth): Trends, Definitions, Standards, and Legislative Revisions

**DOI:** 10.5195/ijt.2023.6599

**Published:** 2023-12-12

**Authors:** Kyrylo S. Malakhov

**Affiliations:** V. M. Glushkov Institute of Cybernetics of the National Academy of Sciences of Ukraine

**Keywords:** Digital health, eHealth, Fundamentals of the Legislation of the Healthcare of Ukraine, Telehealth, Telemedicine, Telerehabilitation, The Strategy for the Development of Telemedicine in Ukraine

## Abstract

**Purpose:**

This article aims to provide an in-depth examination of the digital health system of Ukraine, focusing on the emerging trends, precise definitions, established standards, and recent legislative revisions that shape the practice and implementation of eHealth solutions within the country.

**Background:**

The digital health landscape in Ukraine has witnessed significant transformations, especially in the wake of the COVID-19 pandemic and subsequent military conflicts. These events have catalyzed the expansion of telemedicine services, leading to innovative approaches in healthcare delivery. The national strategy underscores the necessity for human-centric and accessible telemedicine, reinforced by technological neutrality, and harmonization with global standards.

**Methods:**

A review of the current literature, national strategies, and legal documents was conducted, alongside an analysis of data usage and service provision patterns in various Ukrainian regions. Participation in the “Science for Safety and Sustainable Development of Ukraine” competition facilitated project initiatives like the development of a cloud-based platform for patient-centered telerehabilitation for oncology patients.

**Findings:**

The utilization of telemedicine has significantly increased in conflict-affected regions, demonstrating the need for, and the effective deployment of, digital health strategies under crisis conditions. Private health facilities and entrepreneurs have been pivotal in the provision of telemedicine services. Legislative efforts have been geared toward framing telemedicine as an integral component of the national eHealth system, ensuring interoperability, and aligning with international standards and the Internet of Medical Things (IoMT).

**Interpretation:**

The findings underscore the resilience and adaptability of the Ukrainian healthcare system in the face of adversity. There is a clear trend towards a more integrated, patient-focused, and technologically advanced healthcare model, aligning with international trends and prioritizing public health goals over private profits. This progress, however, is contingent upon continuous development, investment in technological infrastructure, and legislative support to sustain and advance digital health initiatives.

The Ukrainian healthcare system, historically characterized by its conventional approach to medicine and patient care, has embarked on a transformative journey into the digital age. The incursion of digital health, also known as eHealth, has emerged as a pivotal aspect of this transformation, reshaping the landscape of healthcare delivery in Ukraine.

In recent years, alongside significant health finance reforms, Ukraine has made strides in advancing digital healthcare solutions, notably an eHealth system and the burgeoning field of digital health, encompassing telemedicine and telerehabilitation. Telemedicine, in particular, offers potential benefits by enhancing efficiency, transparency, and access to quality healthcare services, while concurrently reducing corruption opportunities.

The onset of the full-scale Russian invasion of Ukraine in February 2022, leading to extensive population displacement and targeted aggression against healthcare infrastructure, has underscored the critical role of telemedicine in sustaining healthcare access during crises.

In the public sector, regions such as Odesa, Lviv, and Poltava oblasts (regions) have emerged as leaders in telemedicine adoption, featuring operational telemedicine centers and widespread implementation of doctor-to-doctor consultations. Despite these successes, the scalability of this model in a post-conflict setting, with anticipated limitations on government resources, remains uncertain.

The year 2022 saw a notable uptick in telemedicine utilization in regions affected by active conflict. Notable areas include the Kyiv, Chernihiv, and Sumy oblasts, as well as parts of the Kharkiv, Kherson, and Luhansk oblasts. In these areas, the disruption to traditional healthcare services—owing to population displacement, damage to healthcare infrastructure, and movement restrictions due to safety concerns—necessitated alternative means of delivering medical care. Health professionals adept in telemedicine have endorsed its role in enhancing patient care and facilitating peer consultations for second opinions.

The COVID-19 pandemic further accelerated the adoption of telemedicine as restrictions impeded traditional access to healthcare. The suspension of inter-city and regional transport, coupled with movement restrictions, propelled the use of telemedicine as an alternative to in-person primary and specialized medical care. The response included a grassroots emergence of telemedicine services, with healthcare professionals offering free online consultations through various platforms.

Throughout 2022—2023, telemedicine modalities such as video, medical data sharing, audio, and text consultations, along with telemetry (K. S. Malakhov, 2022; Telehealth in Crisis Situations – The Case of Ukraine, 2022), became increasingly prevalent across Ukrainian healthcare settings. An analysis of telemedicine services relative to the total number of electronic health records reveals that private healthcare facilities and individual practitioners constitute a significantly larger proportion of telemedicine service providers compared to their public counterparts.

In late 2020, the research team of the Institute of Cybernetics, led by scientific supervisor Oleksandr Palagin, became one of the winners of the “Science for Human Security and Society” competition held by the National Research Foundation of Ukraine (The Government of Ukraine, 2023b) with a project called “Transdisciplinary intelligent information and analytical system for the rehabilitation processes support in a pandemic (TISP)” and received a collective grant for its implementation. This project is transdisciplinary applied research aimed at creating a system of information and analytical support for decision-making and developing methodological foundations and practical measures for the comprehensive rehabilitation of members of the society of Ukraine regardless of their health status in a pandemic. At the end of 2021, the project was successfully completed and its scientific and practical results were highly appreciated by the experts of the Foundation. The theoretical results of the research study were published in the form of collective scientific monographs (Chebanyuk & Palagin, 2020; Velychko et al., 2022; Velychko, Petrenko, et al., 2021), and articles in the peer-reviewed journals (Kurgaev & Palagin, 2021; O. Palagin et al., 2021; O. V. Palagin, 2021; O. V. Palagin et al., 2022a, 2022b). The practical results of the project (TISP system) were implemented in various organizations throughout Ukraine (in particular, the department of medical rehabilitation, physiotherapy and sports medicine of Shupyk National Healthcare University of Ukraine). During the implementation of the project, namely, its part related to telerehabilitation and the acquisition by this direction of medicine of transdisciplinary connections with various subject areas that go beyond the modern paradigm of Digital health led to the emergence of the new type of rehabilitation medicine—*hybrid e-rehabilitation medicine*. Core concepts of the hybrid e-rehabilitation notion are presented in (O. V. Palagin et al., 2022a, 2022b, 2023c; Velychko et al., 2022; Velychko, Petrenko, et al., 2021; Telehealth in Crisis Situations – The Case of Ukraine, 2022), and are today expanded by Biomedical Robotics and Bionics technologies. The Biomedical Robotics (Laschowski et al., 2019) research focus area is centered on the design, development, and evaluation of medical robotics systems and smart assistive robotic platforms (Nasr et al., 2021) that enhance the physical capabilities of both patients and clinicians via advancements in mechanical design, modeling and control, sensors and instrumentation, computing, image, and natural language processing (Kurgaev & Petrenko, 1995). Core research topics in this area include medical robotics, haptic interfaces, machine learning, soft robotics, robot-assisted surgery and rehabilitation, tissue modeling, human augmentation, biomechanics, and human-robot interaction. Biomedical robotics research innately draws from several disciplines including mechanical, biomedical and electrical engineering, interactive computing, applied physiology, and materials. Key areas of application and translation include feedback-enabled robotic surgery systems, robot-assisted caregiving, macro-meso-micro-scale image-guided surgical interventions, wearable devices for occupational training and injury prevention, and neuro-integrated prosthetic devices (Laschowski et al., 2022) (also, mathematical modelling and computer simulation of human-exoskeleton systems with energy-efficient actuators, and computer vision and deep learning for autonomous exoskeleton control and decision making during legged locomotion).

As a part of the TISP (Velychko, Petrenko, et al., 2021) the Smart-system for remote support of rehabilitation activities and services (Smart-system)(O. V. Palagin et al., 2022a, 2022b; Velychko et al., 2022; Velychko, Malakhov, et al., 2021; Velychko, Petrenko, et al., 2021) was developed by the research team of the Institute of Cybernetics. For the first time in Ukraine, the implementation of the concept of gaming telerehabilitation was presented. The Smart system has a built-in NLP-Powered set of services for the speed-reading skills evolution in children of primary general education *vec2read* (based on the ideas of Spritz (*Spritz - The Worlds Best Speed Reading App Reading Reimagined*, n.d.)), and *vec2cluster*—a Ukrainian word association game powered by distributional semantics technologies (K. S. Malakhov et al., 2020/2023; O. V. Palagin et al., 2020), and inspired by Google Experiments Semantris. Therefore, the Smart-system can be called the first implemented remote rehabilitation gaming system in Ukraine. The Smart-system also includes various technologies, and web services (O. V. Palagin et al., 2022a, 2022b; Smart-System for Remote Support of Rehabilitation Activities and Services, 2021): remote consulting service, digital doctor's office, service for automated processing and integration of basic e-rehabilitation workflows, collaborative service, *UkrVectōrēs* (K. S. Malakhov et al., 2020/2023) service, *vHealth* electronic library service (Velychko et al., 2022).

At the outset of 2022, our Institute's research team was honored as one of the laureates in the “Science for Safety and Sustainable Development of Ukraine” competition, organized by the Foundation. Our latest endeavor is titled “Development of the cloud-based platform for patient-centered telerehabilitation of oncology patients with mathematical-related modeling” (K. S. Malakhov, 2023a; Telehealth in Crisis Situations – The Case of Ukraine, 2022). This project is committed to constructing a hybrid cloud-based platform and on this foundation, establishing an IT framework for Telerehabilitation Medicine (TMR) aimed at cancer patients. It is designed to serve an extensive range of professionals in Physical Therapy and Rehabilitation Medicine, specifically within the domain of TMR for oncology patients.

The Local Health System Sustainability Project (LHSS), operating under the USAID Integrated Health Systems IDIQ, warrants focused examination due to its substantial contributions to enhancing healthcare accessibility and service quality. The project is actively engaged with partner nations and local entities to mitigate financial obstacles impeding access to healthcare, promote equitable distribution of essential health services to all individuals, and bolster the overall quality of health services provided.

A notable result from this project is its report (USAID, 2023), which summarizes the findings from a USAID LHSS assessment concerning the telemedicine landscape in Ukraine. This evaluation was conducted over the period from September to November 2022, incorporating a comprehensive data collection from nearly every Ukrainian region. The primary objective of this report is to deliver a concise overview of the telemedicine environment to policymakers, developmental collaborators, and various stakeholders, thereby equipping them with the necessary insights to make informed decisions and strategize effectively. The report serves as a valuable resource for understanding the current implementation and challenges of telemedicine in Ukraine, offering a foundation for future policy formulation and healthcare system enhancements.

## Defining Digital Health, Telehealth, Telemedicine, eHealth, and the Internet of Medical Things (IoMT) in the Ukrainian Context

Digital health facilitates optimal and timely healthcare delivery by connecting patients with essential services via advanced telecommunications. This integration encompasses Remote Patient Monitoring (RPM) through diverse monitoring technologies, store-and-forward systems, and mobile health (mHealth) applications. Digital health not only enhances access to healthcare and improves the quality of care but also introduces a level of convenience that is challenging to achieve through traditional in-person consultations. A systematic review in 2018 by Shigekawa et al. (Shigekawa et al., 2018) indicates that digital health interventions for select medical conditions can be as effective as in-person care. The COVID-19 pandemic has accelerated the adoption of digital health elements in hospitals globally, enabling connections between patients and practitioners irrespective of physical presence. Despite this progress and subsequent policy reforms, numerous challenges persist that impede the full-scale and effective implementation of digital health technologies.

The heterogeneity in definitions of ‘digital health’ and ‘telehealth,’ as well as the interchangeable use of distinct terms such as ‘telehealth’ and ‘telemedicine,’ poses a significant challenge in establishing a clear understanding of these concepts (Latifi et al., 2021) This lack of consensus not only leads to confusion among healthcare professionals as they endeavor to provide telehealth services but also complicates the efforts of state and national legislators in crafting effective laws and policies to govern its use. Moreover, inconsistencies in the definition of ‘telehealth’ can result in variable reimbursement policies both globally and at the national level, further complicating the standardization of telehealth practices.

Historically, ‘telemedicine’ has been employed to denote the practice of medicine delivered at a distance. In 2005, a U.S. federal subcommittee acknowledged the importance of both ‘telemedicine’ and ‘telehealth’ as pivotal to enhancing patient care (Doarn et al., 2014). Since then, the lexicon within this field has continued to grow, necessitating precise definitions, particularly in light of the surge in telehealth consultations prompted by the COVID-19 pandemic. There is a general consensus among healthcare organizations and regulatory agencies that ‘telehealth’ and ‘telemedicine’ encompass different scopes. It is commonly accepted that ‘telehealth’ represents the overarching framework, while ‘telemedicine’ refers specifically to the delivery of clinical services within the broader context of ‘telehealth’.

The term ‘telehealth’ has gained prominence in scholarly and clinical discourse. Definitions of telehealth typically refer to the use of technology for healthcare delivery across distances. Beyond direct medical care, some conceptualizations of telehealth encompass a broader array of services within the digital health domain. A survey of academic literature (*Center for Connected Health Policy Fall 2020 Telehealth Report*, 2020; Doarn et al., 2014; Fong et al., 2010; Latifi et al., 2021; Shigekawa et al., 2018) indicates a preference for the term ‘telehealth’ in discussions related to remote healthcare provision. The definitions most frequently cited originate from telehealth-centric organizations and U.S. federal agencies, such as the Department of Health and Human Services (DHHS), Health Resources and Services Administration (HRSA), American Telemedicine Association (ATA), Centers for Medicare & Medicaid Services (CMS), and the American Medical Association (AMA) (Cole et al., 2021; Kruse et al., 2019; Markert et al., 2021; Sasangohar et al., 2018; Tauben et al., 2020; *Telehealth Implementation Playbook*, 2022). Notably, the World Health Organization's (WHO) definition of telehealth is often cited within academic circles and is characterized by its succinctness: ‘Telehealth involves the delivery of health care services, where there is a separation by distance between the patient and the providers. Additionally, some scholars have opted to create composite definitions that draw from multiple institutional sources or present a synthesis of various published definitions (Tauben et al., 2020).

The term ‘Telemedicine’ is frequently utilized in scholarly resources, often characterized as a subset within the broader concept of telehealth. For instance, the Health Resources and Services Administration (HRSA) (HRSA, 2021) delineates ‘telemedicine’ as being confined to the delivery of remote clinical services. This delineation is in contrast to ‘telehealth,’ which is a more encompassing term that includes not only remote clinical services but also non-clinical elements. Non-clinical elements might encompass administrative activities, professional development through provider training, and continuing medical education initiatives. Thus, while ‘telemedicine’ focuses exclusively on the clinical interface between patient and provider across a distance, ‘telehealth’ extends to a wide array of remote healthcare services, underpinning the operational and educational pillars of healthcare institutions.

The WHO provides a comprehensive definition of telehealth (*Global Strategy on Digital Health 2020–2025*, 2021), describing it as the delivery of healthcare services where distance is a critical factor. This service is facilitated by healthcare professionals through the use of information and communication technologies. These technologies enable the valid exchange of information necessary for the diagnosis, treatment, and prevention of disease and injuries, as well as for the research and evaluation purposes, and for the ongoing education of healthcare providers. The objective is to improve the health of individuals and the broader community. However, there is not a universal consensus on the terminology. Some organizations use ‘telemedicine’ and ‘telehealth’ interchangeably. In the context of Ukrainian healthcare, legislation (Verkhovna Rada of Ukraine, 1993) does not distinguish between the two, defining both as an integrated set of actions, technologies, and measures applied to deliver medical care remotely. This encompasses the exchange of medical data in electronic form, including but not limited to electronic messaging and video conferencing. Telehealth in Ukraine is particularly oriented towards ensuring patients receive timely and high-quality medical care, especially in circumstances where time and distance pose significant challenges.

Article 35 of the Law of Ukraine ‘Fundamentals of the Legislation of Ukraine on Healthcare’ (Verkhovna Rada of Ukraine, 1993) delineates the framework within which telehealth services are provided. According to this legislation, telehealth medical care is delivered through various modalities, including telehealth consultations, telehealth councils (conciliums), telemetry, teleconsultations from home, and the administration of medical procedures using electronic and programmatic means. Furthermore, these telehealth services, which may encompass audio and video recordings as well as the documentation of medical equipment readings, are to be conducted in accordance with the protocols established by the central executive authority responsible for public healthcare policy. This authority also specifies the procedural guidelines for the provision of telehealth services.

The term ‘digital health’ is increasingly utilized by organizations and agencies worldwide, sometimes synonymously with ‘telehealth’ and ‘telemedicine’. However, a distinction is often made where ‘telehealth’ is considered a subset within the broader ‘digital health’ framework. The WHO, for example, characterizes digital health as an inclusive category that integrates electronic health (eHealth), mobile health (mHealth), telehealth, and the management of health data, among other elements (WHO, 2020). Digital health offers innovative solutions to enhance health systems, such as facilitating home-based health services, enabling remote care for underserved populations, assisting in the tracking of disease outbreaks, and integrating digital tools to augment the efficiency and responsiveness of healthcare services. In its ‘Global Strategy on Digital Health 2020–2025’, the WHO provides an updated definition: ‘Digital health is the field of knowledge and practice associated with the development and use of digital technologies to improve health’ (*Global Strategy on Digital Health 2020–2025*, 2021). This field extends beyond eHealth, embracing digital consumers and a broader array of smart devices and connected equipment. It also includes emerging and transformative technologies such as the Internet of Medical Things (IoMT), artificial intelligence (AI), big data analytics, biomedical robotics, and bionics. Digital health is a dynamic and extensively researched domain, with IoMT, AI, cloud computing, and wireless sensor networks constituting its core components.

The terms ‘telehealth’ and ‘telemedicine’ often intersect with ‘digital health’, leading to ongoing discussions about whether ‘digital health’ serves as a comprehensive term that goes beyond direct clinical care to include broader healthcare applications. For instance, the U.S. Centers for Disease Control and Prevention (CDC) discusses ‘digital health’ applications on its blog, highlighting an array of measurement technologies. These include personal wearable devices, internal devices, and sensors that have potential utility in health status monitoring, disease diagnosis, and management (Khoury & Dotson, 2021). While the CDC blog recognizes wearable devices that provide continuous patient data as part of digital health, it stops short of explicitly incorporating ‘telehealth’, and ‘telemedicine’ within this category or offering a definitive description of ‘digital health’. Commonly, ‘digital health’ is referenced in literature (*Telehealth Implementation Playbook*, 2022) as an umbrella term that encompasses not only direct patient care interactions but also the broader collection and analysis of health data through technologies such as wearable devices.

In the telehealth lexicon, terms such as ‘asynchronous’, ‘synchronous’, ‘store-and-forward’, ‘remote patient monitoring’, ‘remote physiological monitoring’, and ‘remote therapeutic monitoring’ represent more specialized concepts with generally agreed-upon definitions, offering greater consistency compared to the broader terms of ‘telehealth’, ‘telemedicine’, ‘virtual care’, and ‘digital health’. The term ‘asynchronous’, often associated with ‘store-and-forward’ practices, denotes processes that do not occur in real-time. This modality facilitates flexibility in scheduling, as healthcare providers and patients interact with information at different times and from various locations (*Telehealth Implementation Playbook*, 2022). Although ‘asynchronous’, and ‘store-and-forward’ are frequently used interchangeably, both typically describe the transmission of prerecorded patient data for later review by a healthcare provider. Conversely, ‘synchronous’ interactions involve live, real-time communication between patient and provider, utilizing technology such as video conferencing (*Telehealth Implementation Playbook*, 2022). RPM, a pivotal component of telehealth, has traditionally included devices like glucometers, blood pressure monitors, and scales for patient self-monitoring (Bryant, 2020). The field has expanded to incorporate ‘Internet of Medical Things’ (IoMT) devices, including sophisticated peripheral medical equipment such as digital stethoscopes, otoscopes, and portable ultrasounds, which enable comprehensive remote evaluations (*Remote Patient Monitoring Playbook*, 2022).

The IoMT represents a specialized niche within the broader Internet of Things (IoT) ecosystem, which encompasses a network of interconnected computing devices, mechanical and digital machines, and other entities capable of data exchange without the need for direct human intervention. IoMT can be characterized as (Cardona et al., 2021; França et al., 2021; Hassanien et al., 2021; Hemanth et al., 2021; Syed-Abdul et al., 2021) the convergence of medical devices and applications, including bedside monitors, embedded systems, wearable technologies such as smartwatches, fitness monitors, activity trackers, virtual/mixed reality headsets, implantable devices, and any object capable of sending or receiving health-related data to a remote storage or processing location.

Smart sensors (Hemanth et al., 2021) play a critical role in the IoMT infrastructure by reliably capturing and transmitting vital health indicators, thereby enabling continuous monitoring and assessment of patient health parameters. The strategic integration of IoMT has begun to transform the healthcare industry by bridging significant gaps in the diagnosis, treatment, and ongoing management of patient health. Not only does IoMT facilitate real-time (synchronous) monitoring of patient health, but it also allows for the evaluation of treatment effectiveness. By enabling remote health surveillance, IoMT stands out as a promising solution to enhance patient care, potentially reducing the need for clinical resources and minimizing avoidable hospital admissions.

Remote physiological monitoring is a term that is sometimes used synonymously with remote patient monitoring RPM (*Remote Patient Monitoring Playbook*, 2022). According to various sources (Bryant, 2020; Latifi et al., 2021; *Remote Patient Monitoring Playbook*, 2022) terminologies such as asynchronous, store-and-forward, synchronous, and RPM are all categorized within the scope of ‘telehealth’, and, by extension, ‘telemedicine.’ Additionally, the field of telehealth has introduced ancillary terms such as ‘telehomecare’ and ‘point-of-care’ (POC). The AMA, in its ‘Digital Health Implementation Playbook Series’ (*Remote Patient Monitoring Playbook*, 2022; *Telehealth Implementation Playbook*, 2022) describes POC as a model of remote care that is specifically designed to support individuals with chronic conditions, dementia, or those at an elevated risk of falls, enabling them to continue living in their own homes. This model underscores the flexibility and patient-centered approach that telehealth services aim to provide.

Current research initiatives in Ukraine's digital health and IoMT sectors are marked by the development of advanced information technologies for computerized electrocardiography. Spearheaded by the V.M. Glushkov Institute of Cybernetics of the National Academy of Sciences of Ukraine (*Glushkov Institute of Cybernetics of the NAS of Ukraine*, 2023) (Institute of Cybernetics), one primary objective has been to enhance the diagnostic utility of subtle electrocardiographic signals that typically go undetected during standard analyses (Chaikovsky, 2013). To address this, researchers have formulated an innovative method coupled with software for the detailed scaling of electrocardiograms and the assessment of heart rate variability (Chaikovsky, 2013).

Furthermore, this technology enables ECG examinations to transcend the confines of a clinical setting, facilitated by IoMT devices. It also allows for the transformation of complex analysis results into visuals readily interpretable by both specialists and non-specialists alike. Its application across various conditions, including its integration into a comprehensive screening project in the Khmelnytsky region that encompassed over 22,000 participants (Chaikovsky, Popov, et al., 2021), as well as the analysis of ECG records from the China Kadoorie Biobank (Chaikovsky, Lebedev, et al., 2021), underscores its versatility. This multifunctional technology has proven its value in addressing diverse and significant healthcare challenges through biophysical modeling and practical deployment.

The international patent protections for this technology (Chaykovskyy, 2017; Chaykovskyy et al., 2017) and the interest it has garnered from prestigious institutions like Oxford and Mainz Universities—where it is utilized in extensive population studies—attest to its global relevance. Dr. Illya Chaikovsky, MD, PhD, provides an exhaustive review of this innovative approach to computerized ECG using IoMT devices in (Bocharov et al., 2023; Chaikovsky et al., 2022).

## Exploring the eHealth System in Ukraine: A Detailed Perspective

The concept of ‘eHealth’, or Electronic Health, is understood to have been inspired by the lexicon of the business sector, particularly by the advent of electronic commerce (e-commerce), and its application to various business domains (Eysenbach, 2001). Gerd Eysenbach, a leading figure in this domain, articulates ‘eHealth’ as an emergent discipline situated at the confluence of medical informatics, public health, and business. It encompasses health services and information that are disseminated or augmented via the Internet and associated technologies (Eysenbach, 2001).

Article 3 of the Law of Ukraine ‘Fundamentals of the Legislation of the Healthcare of Ukraine’ (Verkhovna Rada of Ukraine, 1993), alongside ‘The Concept of the Development of Electronic Healthcare in Ukraine’ (Verkhovna Rada of Ukraine, 2020), delineates the term ‘eHealth’ as the collective term for Ukraine's electronic healthcare system, which is functionally synonymous with the ‘digital health system of Ukraine’. This integrated system harnesses information and communication technologies, encapsulating various digital tools and processes. These include Clinical Data Repositories (CDR), Electronic Health Record (EHR) systems, RPM devices, biosensors, and AI-driven algorithms for analytical processing to support decision-making.

The Ukrainian eHealth infrastructure is designed to streamline the management and accounting of medical services and the handling of healthcare data. This is achieved through the creation, storage, publication, and exchange of medical data and documents in electronic formats, including the Master Patient Index and EHRs. At the core of the system is a central database repository that interfaces with multiple Hospital Information Systems (HIS)—also known as hospital management software or hospital management systems—facilitating automatic data interchange via open Application Programming Interfaces (APIs).

**Figure 1.**
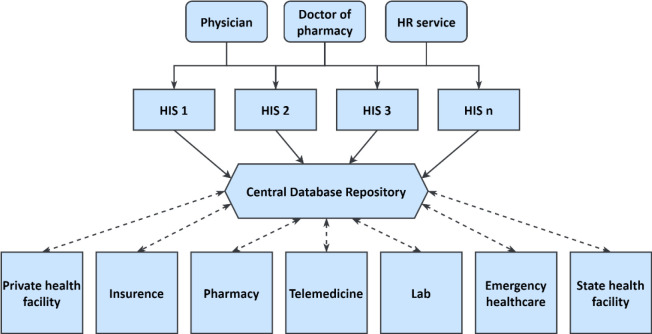
Conceptual eHealth Digital System of Ukraine Architecture

As depicted in Figure 1, the conceptual architecture of Ukraine's eHealth digital system (The Ukrainian Ministry of Health, n.d.) reflects a complex network of interconnected HIS, which currently incorporates over 30 systems. This includes specialized subsystems like Laboratory Information Systems (LIS), Radiology Information Systems (RIS), Picture Archiving and Communication Systems (PACS), and an array of medical hardware and software solutions.

Ukraine's eHealth system is architecturally modular, designed to cater to the diverse needs of its users. Each module encompasses a distinct set of functionalities tailored to the requirements of different stakeholders within the healthcare ecosystem. For instance, a healthcare facility may adopt an administrative module from one HIS, while physicians at the same facility might use a ‘Primary care physician's digital workplace’ module from another HIS. The modular approach allows for flexibility and specificity in user engagement with the eHealth system. As of the current status (The Ukrainian Ministry of Health, n.d.), the eHealth system offers the following modules for integration:

- *Primary Healthcare Provider Administration Module.* This module facilitates contractual engagements with the National Health Service of Ukraine (NHSU) and manages funding allocations for primary healthcare institutions.
- *Primary Care Physician's Digital Workplace*. It provides primary healthcare physicians with tools for handling declarations on physician selection, managing EHRs, and processing electronic prescriptions within the Affordable Medicines reimbursement program.
- *Pharmacy Administration Module.* This component is responsible for the registration of pharmacies and their affiliates, pharmacists, and the execution of reimbursement contracts with the NHSU.
- *Pharmacist's Digital Workplace.* Designed for pharmacists, this module aids in processing electronic prescriptions under the Affordable Medicines reimbursement program.
- *Specialized Healthcare Provider Administrative Module.* It includes tools for registering specialized healthcare institutions, their divisions, and user management.
- *Specialized Healthcare Physician's Digital Workplace:* This module is equipped with functionalities for managing EHRs, generating diagnostic reports, and scheduling electronic appointments, including for patients who are not yet identified.
- *EHR Managing Module.* It oversees the management and processing of EHRs for both identified and unidentified patients, including patient verification and the consolidation of records for unidentified patients with those of identified ones.
- *Data Access Module.* Provides comprehensive access to all registries maintained by the eHealth system, ensuring that authorized users can retrieve necessary data as required.

These modules collectively enhance the operability of the eHealth system, ensuring that it can accommodate a broad spectrum of healthcare activities ranging from administrative operations to direct patient care.

The foundational framework for a ‘digital workplace’, also referred to as the Research & Development Workstation Environment (RDWE), along with its core concepts, design principles, requirements, and comprehensive information model, was systematically delineated in research studies conducted by our team in 2014 (O. V. Palagin et al., 2014) and 2018 (O. V. Palagin et al., 2018).

Despite the economic challenges and the imposition of martial law due to the Russian full-scale invasion, Ukraine's professional healthcare community—including the Ukrainian Ministry of Health, the government organization EZdorovya—State-Owned Enterprise eHealth, and select experts from the nation's scientific and healthcare sectors—is actively developing a comprehensive Concept/Strategy for telemedicine expansion and an accompanying Action Plan for its implementation. Within the Interdepartmental Ukrainian Working Group, the technical and architectural aspects of telemedicine are being addressed by an expert subgroup—in accordance with the Order of the Ukrainian Ministry of Health “On the Interdepartmental Working Group on the Development of the Concept of Telemedicine Implementation” of February 11, 2022, No. 281 (Verkhovna Rada of Ukraine, 2022).

The proposed concept/strategy delineates several key priorities for the advancement of telemedicine, including:

- *Accessibility Enhancement.* Facilitating access to telemedicine services by integrating them within the eHealth framework and leveraging other information and communication systems, including mobile applications and bespoke telemedicine solutions.
- *Patient Empowerment.* Broadening patient access to personal medical records, thereby fostering greater patient engagement in their healthcare management.
- *Direct Telemedicine Engagement.* Enabling patients to initiate telemedicine consultations, thus streamlining the interaction process with healthcare providers.
- *Data Interoperability.* Guaranteeing the seamless integration of patient medical records, ensuring that data remains accessible and consistent across different technological platforms and storage locations.
- *Quality and Safety Assurance.* Maintaining high standards of quality and safety within telemedicine practices to protect patient welfare.
- *Regulatory Framework Enhancement.* Refining the legal and regulatory structures to support the expansion and efficacy of telemedicine services.
- *Public Engagement and Education*. Promoting the benefits and knowledge of telemedicine through targeted media campaigns, educational initiatives, and other outreach activities.

In the wake of the full-scale Russian invasion, the operational landscape for telemedicine services has been significantly altered. The enactment of martial law has necessitated adaptations to the provision of medical and rehabilitation services. Consequently, medical practitioners and rehabilitation specialists, irrespective of their nationality, are now authorized to deliver medical and rehabilitative care via telemedicine, contingent upon their registration within the requisite information and communication systems (Telemedicine issues under martial law are regulated by the Orders of the Ukrainian Ministry of Health dated June 20, 2022, No. 1062 “On the organization of medical care using telemedicine under martial law” and September 17, 2022, No. 1695 “On approval of the Procedure for the provision of medical care using telemedicine, rehabilitation assistance using telerehabilitation for the period of martial law in Ukraine or its individual areas.”).

Mr. Kyrylo Malakhov (MSc in Computer Science & IT, Bachelor of philology—English and foreign literature, Researcher, Backend developer, DevOps engineer, Microprocessor Technology Lab, Glushkov Institute of Cybernetics of the National Academy of Sciences of Ukraine) (K. S. Malakhov, 2023b), and Dr. Oleksandr Palagin (Academician of the National Academy of Sciences of Ukraine, DSc, Professor, Deputy Director, Head of Microprocessor Technology Lab, Glushkov Institute of Cybernetics of the National Academy of Sciences of Ukraine) are members of this subgroup, are contributing to the formulation of core components crucial for the integration of contemporary Electronic Health Record (EHR) standards into Ukraine's eHealth system. These standards include Health Level 7 Fast Healthcare Interoperability Resources (HL7 FHIR), Health Level 7 Clinical Document Architecture (HL7 CDA), the openEHR Archetype Object Model and Archetype Definition Language (ADL), Logical Observation Identifiers Names and Codes (LOINC), and the World Health Organization International Classification of Functioning, Disability, and Health (WHO ICF). These efforts are pivotal in ensuring that Ukraine's eHealth system aligns with international best practices for interoperability and comprehensive health data management (Conceptual Models for Clinical Data Repository Implementation, 2022).

The establishment of foundational telemedicine frameworks in Ukraine is the culmination of a concerted effort involving key stakeholders. This collaborative network includes the Government of Ukraine, the Ministry of Health, the National Health Service of Ukraine, the state-owned enterprise EZdorovya—eHealth, and the Interdepartmental Working Group on the Development of the Concept of Telemedicine Implementation in Ukraine, as well as additional entities within the Ukrainian professional healthcare community. The synergy of these organizations has yielded two pivotal documents that are set to shape the trajectory of telemedicine within the nation:

- *The Strategy for the Development of Telemedicine in Ukraine*—a comprehensive plan outlining the vision, objectives, and actions for the integration and advancement of telemedicine services. The order of the Government of Ukraine dated July 14, 2023, No. 625-p, titled “On approval of the Strategy for the Development of Telemedicine in Ukraine” (The Government of Ukraine, 2023a).
- *Amendments to Specific Legislative Acts of Ukraine Pertaining to the Functioning of Telemedicine* (Verkhovna Rada of Ukraine, 2023)—legislative revisions aimed at enhancing the legal and regulatory framework for the effective functioning of telemedicine. The Law of Ukraine dated August 9, 2023, No. 3301-IX, titled “On Amendments to Specific Legislative Acts of Ukraine Pertaining to the Functioning of Telemedicine.”

These documents represent significant milestones in the standardization and expansion of telemedicine services, reflecting a unified approach to healthcare modernization.

## The Law of Ukraine No. 3301-IX “On Amendments to Specific Legislative Acts of Ukraine Pertaining to the Functioning of Telemedicine”

*This section provides an English rendition of the Law of Ukraine, dated August 9, 2023, No. 3301-IX, titled “On Amendments to Specific Legislative Acts of Ukraine Pertaining to the Functioning of Telemedicine.” It is imperative to acknowledge that this English translation is not sanctioned as an official legal document. It has been prepared exclusively for informational purposes (and may not represent the legal nuance of the original Ukrainian language document) to aid the understanding of international health professionals regarding Ukraine's legislative developments in telemedicine.*

### The Verkhovna Rada of Ukraine decrees:

- To make amendments to the following legislative acts of Ukraine:
  1. In the Fundamentals of the Legislation of the Healthcare of Ukraine (Bulletin of the Verkhovna Rada of Ukraine, 1993, No. 4, Article 19 with subsequent amendments) (Verkhovna Rada of Ukraine, 1993):
    1) in the first part of Article 3:
      redefine the term “telemedicine” as follows:
      “telemedicine—a complex of actions, technologies, and measures applied to provide patients with medical and/or rehabilitation assistance through telemedicine methods and means in a remote manner, which is a component of electronic healthcare”;
      add the following terms in alphabetical order with the content:
      “electronic healthcare (eHealth) – a system of mutually acceptable information relations of all subjects in the healthcare sector based on the use of methods, measures, and technologies in a digital environment, including information and communication technologies aimed at supporting the healthcare sector, including medical services, preventive health monitoring and promotion, improvement of the quality and longevity of the population, medical literature and education in the field of healthcare, knowledge and research, use of digital services for obtaining necessary data, knowledge, and skills to provide medical and/or rehabilitation assistance, performance of operational functions of the public health system”;
      “telemedicine tool (telemedicine means) – any technical and software tool and/or other component of the information (automated) system for providing patients with medical and/or rehabilitation assistance using telemedicine”;
      “telemedicine method – the order of actions using technical and software tools and/or other components of the information (automated) system, which in complex interaction provide patients with medical and/or rehabilitation assistance using telemedicine”;
      “telediagnosis – conducting diagnostic procedures using information and communication technologies for the exchange of medical data, which includes, among other things, the transmission of images, test results, other examinations, data from medical equipment, and any documents related to health”;
      “teleconsultation (tele-video consultation) – communication (interaction) between two or more participants (medical (pharmaceutical) workers and/or rehabilitation specialists and patients) using information and communication technologies for the purpose of providing patients with medical and/or rehabilitation assistance and prevention”;
      “telemedicine network – a component of electronic healthcare, which constitutes a set of telemedicine tools and methods, medical service providers, and organizational and technical measures for effective interaction between medical workers, rehabilitation specialists, and patients”;
      “telemetry – a set of technologies and means that allow for the remote measurement of a patient's health status indicators, as well as processing and transmitting information/data about such indicators”;
      “digital competence of healthcare workers – the ability to confidently, professionally, and responsibly use digital technologies in professional activity, as well as for continuous professional development with the aim of achieving healthcare goals, organizing and providing medical and/or rehabilitation assistance to the population, which includes, in particular, digital and information literacy, digital communication and collaboration, use of digital technologies, cyber hygiene, and cybersecurity”;
    2) in Article 4:
      the seventh paragraph should be presented in the following edition:
      “focus on modern health standards, medical and rehabilitation assistance, application of modern digital technologies, telemedicine and telerehabilitation, combining national traditions and achievements with the best global experiences in the field of healthcare”;
      supplement with eleventh and twelfth paragraphs of the following content:
      “establishing a unified medical data space as a combination of databases, their management and usage technologies, and information and communication systems that operate on the basis of unified principles and general rules, as well as on the principles of interoperability, integration, and implementation of electronic healthcare tools;
      adherence to the principles of accessibility and inclusion in the provision of medical and/or rehabilitation assistance, including the use of telemedicine methods and means”;
    3) Item “d” of the first part of Article 6 should be presented in the following edition:
      “d) qualified medical and rehabilitation assistance ensuring an adequate level of personal data protection, including the free choice of healthcare institution, choice of doctor and/or rehabilitation specialist, choice of treatment and/or rehabilitation methods according to the recommendations of the doctor and/or rehabilitation specialist, and the possibility of receiving such assistance throughout the territory of Ukraine”;
    4) Article 35^6^ shall be presented in the following edition:
      “**Article 35^6^**. Provision of Medical and/or Rehabilitation Assistance Using Telemedicine.
      Medical and/or rehabilitation assistance using telemedicine implies the possibility of providing patients with medical services using telemedicine methods and tools, employing data, electronic communication, and software-hardware infrastructure, the means of which enable interaction between the patient and medical workers and/or rehabilitation specialists.
      Medical and/or rehabilitation assistance using telemedicine is provided with the aim of ensuring timely access for patients to medical and/or rehabilitation assistance, disease prevention, diagnostics, observation, health condition monitoring, treatment, and includes the possibility of providing patients with medical services using telemedicine methods and tools.
      The provision of medical and/or rehabilitation assistance using telemedicine is carried out by healthcare institutions or individual entrepreneurs who are registered and have obtained a license to conduct medical practice according to the procedure established by law.
      The specifics of applying telemedicine methods and tools when providing emergency, primary, specialized, and palliative care are determined by the central executive body that ensures the formation and implements state policy in the field of healthcare.
      The provision of medical and/or rehabilitation assistance using telemedicine is performed through teleconsultation (tele-video consultation), telediagnostics along with examination, telerehabilitation, and the application of other methods that do not contradict the law, by exchanging personal data, medical data, medical diagnostic data in electronic form between a medical worker and/or rehabilitation specialist and a patient, or between medical workers and/or rehabilitation specialists.
      The provision of medical and/or rehabilitation assistance using telemedicine is carried out in compliance with the requirements for maintaining medical confidentiality and the confidentiality of information about the patient's health condition, in accordance with the requirements of the laws of Ukraine “On Information”, “On Protection of Personal Data”, “On Protection of data in Information and Communication Systems”, as well as in compliance with the norms of ethics and deontology of medical assistance provision.
      The procedure for providing medical and/or rehabilitation assistance using telemedicine is determined by the central executive body that ensures the formation and implements state policy in the field of healthcare”;
    5) Items “a”, “g”, and “d” of the first part of Article 78 shall be presented in the following edition:
      “a) to facilitate the protection and strengthening of people's health, the prevention and treatment of diseases, and to provide timely qualified medical, pharmaceutical, and rehabilitation assistance, including using methods and means of telemedicine”;
      g) to continuously improve the level of professional knowledge and skills, including the level of digital competence”;
      “d) to provide consultative assistance to their colleagues and other healthcare workers, rehabilitation specialists, including using methods and means of telemedicine”;
    6) Sub-item 3 of point 2 of Section XIII “Final and Transitional Provisions” shall be presented in the following edition:
      “3) as an exception from Article 74 of these Fundamentals, medical workers, rehabilitation specialists who are foreigners (except citizens of the Russian Federation or the Republic of Belarus) or stateless persons who have come to Ukraine to provide medical, rehabilitation assistance on a voluntary basis at the invitation of a healthcare institution or other enterprise, organization, or institution that involves foreigners and stateless persons in volunteer activities, may be engaged in providing medical assistance, rehabilitation assistance if such medical workers, rehabilitation specialists meet the requirements for education and professional qualifications and have documents on education and appropriate professional qualification, certified by consular legalization or by affixing an apostille in the country where they work. The healthcare institution or other enterprise, institution, or organization that has engaged foreigners or stateless persons who meet the requirements for education and professional qualifications and have documents on education and appropriate professional qualification, certified by consular legalization or by affixing an apostille in the country where they work, shall inform the central executive body that ensures the formation and implementation of state policy in the field of healthcare about the term for which the said persons are engaged, within five working days from the moment of involvement. Medical workers, rehabilitation specialists who are foreigners (except citizens of the Russian Federation or the Republic of Belarus) or stateless persons may be engaged in providing medical assistance, rehabilitation assistance using telemedicine and telerehabilitation, provided they are registered in the information and communication system that ensures the provision of medical and rehabilitation assistance using telemedicine and telerehabilitation. Information and communication systems whose rights are registered in the Russian Federation or the Republic of Belarus may not be used for providing such assistance”.
  2. In the Law of Ukraine “On Rehabilitation in the Field of Healthcare” (Bulletin of the Verkhovna Rada of Ukraine, 2021, No. 8, Article 59 with subsequent amendments) (Verkhovna Rada of Ukraines, 2021):
    1) The nineteenth paragraph of the first part of Article 1 shall be presented in the following edition:
      “telerehabilitation – a component of telemedicine that provides patients with rehabilitation assistance by rehabilitation specialists through teleconsultation (tele-video consultation) along with examination, telemetry, and other forms that do not contradict the legislation, using information and communication technologies”;
    2) Article 19 shall be presented in the following edition:
      “**Article 19.** Provision of Rehabilitation Assistance Using Telerehabilitation.
      1. The provision of rehabilitation assistance using telerehabilitation is carried out in accordance with the Fundamentals of the Legislation of Ukraine on Healthcare, this Law, and other legislative acts regulating health-related matters”;
    3) Sub-item 1 of point 2 of Section V “Final and Transitional Provisions” shall be excluded.
- Final Provisions
  1. This Law shall come into force the day after its publication.
  2. Within three months from the date this Law comes into effect, the Government of Ukraine shall:
    bring their regulatory acts into compliance with this Law;
    ensure that the ministries and other central executive bodies bring their regulatory acts into compliance with this Law.

## The Order of the Government of Ukraine No. 625-p “On approval of the Strategy for the Development of Telemedicine in Ukraine”

*This section introduces an English translation of the Government of Ukraine's Order No. 625-p “On Approval of the Strategy for the Development of Telemedicine in Ukraine.” It must be emphasized that this English translation, rendered from the original Ukrainian, does not constitute an official document. It is provided exclusively for the informational use of a broad international audience of health professionals. The translation is selective, encompassing principal sections of the strategy that, in the translator's judgment, are sufficient to convey the essence of the document.*

### Description of the Problems that Necessitated the Adoption of the Strategy, and of the Normative Legal Acts Currently in Effect in the Relevant Field

Telemedicine must be considered as a tool for comprehensive electronic health care and the digital transformation of Ukraine. Telemedicine technologies should serve as effective tools for providing access to medical care, health restoration, rehabilitation, prevention, communication, science, and education, and expand the possibilities of receiving medical care beyond the borders of Ukraine.

The current state of telemedicine development in Ukraine does not meet the needs and challenges of today due to the lack of directions for the development of telemedical technologies in Ukraine as an integral component of electronic healthcare, which leads to the existence of such problems in this field.

*Limited access to medical services and medical data.*

Patients' access to medical services is limited due to destroyed medical and transport infrastructure amid armed aggression, and the application of telemedicine is complicated by the limited availability of unified medical tools, including patient offices, technical applications, and the ability to schedule telemedical consultations.

The comprehensive realization of citizens' rights to healthcare is impossible without access to and management of their own medical data. In the current state of Ukrainian healthcare, access to the majority of diagnostic data after examinations is difficult for citizens and medical professionals, with some data being lost due to organizational and technical reasons. This results in the need for repeated diagnostics, leads to unjustified time expenditures by medical workers and citizens, and complicates the ability to track dynamic changes in health indicators.

Thus, one of the key problems is the insufficient collection and transmission of medical diagnostic data about the patient's health status to the patient's electronic health record within the Ukraine's electronic healthcare system – eHealth system. The results of the application of telemedicine in providing medical assistance to the patient are recorded in a limited scope in the electronic health record because telemedicine technologies are not integrated with the eHealth system. The lack of a more comprehensive set of diagnostic data at different stages of medical care can lead to a decrease in the quality of care, especially in emergency situations where time is critically important.

*Insufficient technical provision for telemedicine.*

There is a lack of a unified architecture for the application of telemedicine, and the technical requirements for its interaction with the Central Database Repository (CDBR) of the eHealth system have not been defined;

the communication infrastructure in healthcare facilities is underdeveloped;

there are no compatibility rules and insufficient standardization requirements for devices and technologies used in the field of telemedicine;

there is no systematic approach to the technical implementation of telemedicine.

*There are no quality standards for providing medical assistance using telemedicine.*

The medical services that may employ telemedicine have not been defined, and there are no restrictions on its use for safety and appropriateness for the patient;

the current procedure for providing medical assistance via telemedicine does not take into account the needs of patients and modern conditions;

conditions for the use of telemedicine have not been included in the standards for providing medical assistance;

there are no quality requirements and evidence-based clinical protocols that include the provision of medical assistance using telemedicine;

the model of patient interaction with a doctor in the provision of medical assistance using telemedicine has not been defined;

a mechanism for quality control of medical assistance provided using telemedicine has not been developed.

*Normative legal acts regulating the application of telemedicine have gaps.*

The main normative legal framework regarding telemedicine was established before 2017. The current regulations do not account for the changes that have occurred due to healthcare reform, in particular changes in the medical services financing system and the implementation of the eHealth system, as well as modern challenges and new service conditions caused by the pandemic and war:

outdated terminology is used;

there is no normative regulation regarding the requirements for integration with the eHealth system and Information and Communications Systems (ICS), which use telemedicine methods.

*Insufficient organizational and resource support for telemedicine.*

The absence of clearly defined goals, tasks, processes, a development plan for telemedicine, and the delineation of areas of responsibility leads to uncoordinated actions, duplication, and fragmentation of efforts by interested parties – the state and private sector, donors, and international technical assistance projects. This slows down the development of telemedicine and minimizes the effect of investments made over the last decade.

There is a lack of clear understanding of the extent to which telemedicine expenditures should be included in state and local budgets for healthcare funding. International experience indicates a similar problem in many countries, where limited available resources and uncoordinated mechanisms for reimbursement of costs for providing medical assistance using telemedicine are significant barriers to the initiation of such assistance and its further financial support.

Although telemedicine is not a separate medical specialty, the application of telemedicine methods requires appropriate personnel training. At the same time, the system of pre-graduate and postgraduate medical education predominantly does not include modern educational programs in telemedicine, and existing programs need review and improvement.

At the level of postgraduate education, educational programs in telemedicine are chosen by a small number of medical workers. Mostly, such training is funded by donors or as part of a technical support package provided by suppliers of telemetry, teleradiology, other equipment, and operators of Hospital Information Systems (HIS). Other problems include the low level of computer literacy among medical workers and a shortage of technical specialists to ensure the support of telemedicine technologies in healthcare institutions.

### Strategic Objectives and Indicators of Their Achievement

The goal of the Strategy is to form and define the directions, principles, and mechanisms for the development of telemedicine to preserve and strengthen the nation's health by improving the quality and accessibility of medical services and increasing the efficiency of healthcare resource utilization.

The implementation of the Strategy is envisaged along the following directions:

*ensuring patient access to medical services, protection of their rights and interests when receiving medical care, namely*:

expanding patients' access to their own medical data and to electronic services that can be implemented at the stages of telemedicine development;

initiating telemedical interaction by citizens, broadening their opportunities to independently choose the method of receiving medical care using telemedicine, including telemedical consultation;

enhancing the functionality of the CDBR of the eHealth system regarding the processing of results from the use of telemedical methods in the provision of medical services;

*development of the technical provisions for telemedicine, namely:*

*creation of a technical architecture for telemedicine with consideration of such approaches:*

formation and development of telemedicine as an integral component of the eHealth system based on the principles of openness, transparency, accessibility, and universality;

harmonious expansion of the service-oriented hierarchical architecture of telemedicine, consisting of vertical levels (unified rules and interaction with the eHealth system) and horizontal levels (compatibility of technical solutions and linkage between HISs and other ICSs);

establishment of a unified telemedicine network of diagnostic equipment and hardware-software solutions for video conferencing within the framework of primary methods: *teleconsultation, biotelemetry, teleradiology*;

implementation of centralized hardware-software solutions at the national level for the development of infrastructure for the collection, exchange, and analysis of medical data;

ensuring the interoperability of telemedicine hardware-software solutions by implementing standards and protocols for automated data exchange;

developing new or modifying existing functional processes of telemedicine, adapted to interactions between the CDBR of the eHealth system and its peripheral component through an open application programming interface;

development and integration with the eHealth system of networks of telemetric mobile diagnostic complexes, tele-screening accessories, and medical devices used for diagnostics;

*implementing proper technical regulation, standardization, and structuring of telemedicine interactions and data, namely:*

implementing integrated telemedicine hardware-software solutions that provide a unified and standardized process for the exchange of EHRs or links to diagnostic data;

harmonizing national standards with current international standards and classifiers for further integration and data exchange with the international telemedicine space;

implementing standards for unified audio and video communication systems for conducting quality and secure teleconsultations in real time;

encouraging the development of telemedicine technologies and tools designed for various categories of users, in accordance with the fundamental principles and rules of the eHealth system;

ensuring the accessibility of telemedicine through HISs and other ICSs, mobile applications, and specialized telemedicine solutions according to the nature of medical services;

defining a list of telemedicine data for further processing in the CDBR of the eHealth system;

implementing quality and safety standards for software solutions and equipment, cybersecurity of the telemedicine process, controllability, and data protection in ICSs;

creating technical conditions for compatibility, exchange, storage, and use of medical data through unified standards and protocols used during the provision of medical assistance using telemedicine;

*development of the information and communication infrastructure, telemedicine network, and data storage systems, namely:*

ensuring patient diagnostic data accessibility regardless of the technological solutions and storage location;

using existing secure communication channels to provide medical assistance using telemedicine;

expanding the distributed technical infrastructure for medical data processing as necessary for optimal telemedicine process support;

ensuring the operation, maintenance, and administration of an automated system for storing and exchanging digital diagnostic data in the CDBR of the eHealth system obtained through telemedicine;

defining technical requirements for telemetric mobile diagnostic complexes, tele-screening accessories, and medical devices used for diagnostics in biotelemetry, telemedical visualization, etc.;

rational and optimal use of asynchronous and synchronous methods of telemedical consultations, and diagnostics for the best outcomes;

optimal utilization of digital diagnostic equipment integrated into the information and communication network for exchanging and storing medical data, particularly radiological images, as well as for managing diagnostic resources available in the country;

modernizing and expanding the information and communication infrastructure in line with global trends in the digital transformation of healthcare. For instance, by the order of the Government of Ukraine dated December 28, 2020, No. 1671, the Concept for the Development of Electronic Healthcare was approved, aimed at creating conditions and foundations for the development of electronic healthcare in Ukraine in general and the eHealth system in particular, i.e., an ecosystem of information relations of all participants in the state's medical environment, based on economically efficient and safe use of information and communication technologies. The first phase of the Concept's implementation (2020–2022) ensured the use of advanced international medical and data standards, and the second phase (2023–2025) plans the continuation of integration into the global medical data space. Additionally, within the EU4Digital eHealth network (network for connecting healthcare systems in the EU and Eastern neighboring countries), Ukraine participates in the implementation of guiding principles and standards of electronic healthcare, particularly telemedicine. Thus, in the architecture of the national eHealth system, the FHIR protocol and other solutions have been applied, which are an object-oriented standard in healthcare and a prerequisite for harmonious integration into the European medical information ecosystem. General policies and decisions based on key normative legal acts of the national electronic healthcare are directed towards integration into the European community;

*ensuring proper quality and safety of telemedicine, including telemedicine tools, software, and medical data exchange, namely:*

defining medical services that employ telemedicine tools or, conversely, are restricted due to patient safety considerations;

updating protocols for providing medical assistance to patients taking into account the capabilities of telemedicine;

implementing specialized software solutions for the exchange, storage, and interpretation of digital images;

applying defined means of identification and authentication for participants in the telemedicine process;

creating technical and legal conditions for access to anonymized aggregated medical data and transparent rules for their use;

ensuring the possibility of using telemetric diagnostic systems at all levels of medical assistance, based on clinical appropriateness in the interest of the patient;

adherence by medical workers to safety rules when providing medical assistance using telemedicine tools;

*improving organizational, financial, and staffing provisions for telemedicine, namely:*

creating a conducive environment to stimulate the development of a free market for telemedicine tools and technologies;

identifying priority investments in the implementation of technologies and personnel training;

developing approaches for the procurement of medical services using telemedicine within the framework of the medical guarantees program (including requirements for providers and services, conditions for contract conclusion, payment mechanisms, etc.) to integrate priority and evidence-based telemedicine methods into medical services reimbursed by the state;

implementing and supporting investment projects aimed at the development of providing medical assistance using telemedicine, technologies, and technical infrastructure of healthcare institutions, owner and administrator of the eHealth system;

applying mechanisms of public-private partnership;

assessing the needs for technical personnel training according to the scope and scale of telemedicine development;

updating educational programs, enhancing qualifications, conducting trainings (including online) for medical and technical workers regarding the application of telemedicine tools and methods;

carrying out media and educational outreach to support the expansion and popularization of telemedicine.

To achieve the objectives of the Strategy, an Operational Plan for the implementation of the Strategy for the Development of Telemedicine in Ukraine for 2023–2025 is approved, which defines tasks and measures, implementers, stages, and performance indicators.

Telemedicine should not be seen as a replacement for traditional methods and ways of providing medical assistance. The introduction of digital technologies into healthcare must be based on societal goals, not private profit. The modern capabilities of digital technologies and data processing should be used to support public health missions and ensure universal coverage of medical assistance to the population, enhancing its accessibility and quality, and fulfilling national security tasks considering clinical and economic appropriateness (quality and benefits corresponding to cost).

*The development of telemedicine is envisioned to be based on the following principles:*

*Human-centricity:* At the core of healthcare is the individual, and accordingly, any method, technology, influence, and consequences of telemedicine are considered in terms of benefit, safety, convenience, and adherence to citizens' rights. The implementation of this principle in national healthcare should become the foundation for the development of telemedicine in the country;

*Accessibility of medical assistance:* Telemedicine expands the population's access to medical assistance, including for residents of remote communities, people with physical disabilities and chronic diseases, internally displaced persons, and citizens who have gone abroad under war conditions, guided by the principle that everyone should have access to safe, quality, and evidence-based medical assistance wherever they are;

*Interaction with the eHealth system, interoperability:* The interconnection of HISs and other ICSs with each other and with the CDBR of the eHealth system to ensure compatibility and universal access to medical information obtained by telemedicine methods;

*Deepening digital transformation:* Modernization, integration, and expansion of information and communication infrastructure, ensuring open, transparent, and sustainable functioning of application software systems in the eHealth ecosystem;

*Protection and security:* Compliance with software and equipment requirements to ensure cybersecurity and data protection in HISs and other ICSs when providing medical assistance using telemedicine;

*Openness and transparency:* Creating conditions for citizens' awareness of the existence of information registration systems, databases, and the possibility of accessing them, about the volumes and content of data, and the purposes of their use;

*Technological neutrality and independence from hardware-software solution providers:* Technological solutions must conform to modern international standards, with a priority on development using open application programming interfaces, and minimal risks of dependence on telemedicine solution providers;

*Internet of Medical Things:* Telemedicine has a positive impact on public health because it allows optimal and safe use of personal communication devices, thereby promoting the rational use of technical and personnel resources in healthcare, and creates conditions for citizens to take responsible care of their own health;

*Modernity and alignment with global standards and trends:* The development of telemedicine around the world reflects global technological and informational progress. Each year sees accelerated updates of technologies and data processing methods, devices are becoming smaller, their computational power is increasing, and new ways of transmitting data remotely are emerging. Therefore, the development of telehomecare must be updated in line with global trends and adopt cutting-edge technologies.

The current assessment of the Strategy's implementation results is conducted annually by the Ukrainian Ministry of Health and is made public.

Reports on the effectiveness of the Strategy's implementation are published on the official website of the Ukrainian Ministry of Health.

## Conclusion

The evolution of Ukraine's digital health system, particularly eHealth, amidst its challenging socio-political landscape, is a testament to the nation's commitment to advancing healthcare delivery through innovative means. The strategic adoption of telemedicine has proven essential in ensuring continuous patient care during times of crisis, whether due to the COVID-19 pandemic or the exigencies of military conflict. The establishment of a patient-centered telerehabilitation platform for oncology patients marks a significant step towards specialized care, demonstrating the potential of cloud-based solutions and mathematical modeling in enhancing the scope and quality of healthcare services.

The collaborative efforts of the Interdepartmental Working Group on the Development of the Concept of Telemedicine Implementation in Ukraine have played a crucial role in navigating the complexities of this digital transformation. These efforts are supported by the annual assessments and transparent reporting by the Ministry of Health, which are fundamental for gauging progress and identifying areas for improvement.

The legislative revisions, while still in the process of refinement, are indicative of a robust attempt to create an interoperable, secure, and comprehensive digital health infrastructure. These laws are instrumental in providing a framework that protects patient data, allows for the seamless integration of technological advancements, and aligns with international health standards.

The increased reliance on telemedicine services in private healthcare facilities and by private entrepreneurs reflects a broader trend towards decentralized healthcare. This shift is driven by the necessity to adapt to the limitations imposed by the current geopolitical situation and the global health crisis.

Ukraine's strategic approach to telemedicine, underpinned by principles of human-centricity, accessibility, interoperability, and alignment with global trends, sets a framework that other nations might learn from or adapt. The nation's journey through digital health adaptation, amidst adversity, offers valuable insights into the role of policy, technology, and collaboration in shaping the future of healthcare systems worldwide.

As Ukraine continues to navigate through periods of turbulence, the resilience of its healthcare system through digital health innovations stands as a beacon of progress. The ongoing commitment to technological neutrality, the Internet of Medical Things, and the adherence to global standards ensures that the trajectory of Ukraine's eHealth system is not only responsive to current needs but also poised for future advancements.

## Further Research

Subsequent research endeavors will focus on advancing the components of a system designed to support the comprehensive cycle of scientific research within the realm of digital health, specifically within the Ukrainian eHealth system. A pivotal element of this ongoing research is the development of an AI Assistant aimed at augmenting the capabilities of physical rehabilitation and telerehabilitation therapists, students, and patients. This initiative is encapsulated in the project known as *medlocalgpt*, which fuses intelligent data analysis with computational linguistics to enhance digital health services.

The *medlocalgpt* project is distinctive for its integration of advanced information technology that synergizes large language models (LLMs) (O. V. Palagin et al., 2023a), word embeddings models (O. V. Palagin et al., 2020), and aspects of ontology engineering (K. S. Malakhov et al., 2023; A. Litvin et al., 2023; O. V. Palagin et al., 2023b, 2023c). This includes the utilization of SPARQL or SQL for precise database queries, facilitating the retrieval of fully structured information, and the innovative application of prompt tuning to LLMs, incorporating selected domain-specific knowledge. This project employs a refined Retrieval Augmented Generation method to improve the interaction between AI systems and end-users.

An initial preview of the *medlocalgpt* project is accessible through a public GitHub repository (K. S. Malakhov, 2023/2023), allowing for community engagement and collaboration. The project stands as a testament to the dynamic intersection of AI, language processing, and healthcare, promising to yield tools that will significantly enhance the digital health landscape

## Author Note

MSc in Computer Science & IT, Bachelor of Philology—English and foreign literature, Researcher, Backend developer, DevOps engineer, Microprocessor Technology Lab, Glushkov Institute of Cybernetics of the National Academy of Sciences of Ukraine. Graduate of Luhansk Taras Shevchenko National University.
